# Progress in Medicine

**Published:** 1897-06-12

**Authors:** 


### Progress in Medicine.

### FEVERS.

*(Continued from page 146.)*

**Typhoid.**—An important paper on the separation and identification of the typhoid bacillus and on the existence of many definite species of the bacillus coli is due to F. W. Stoddart.[1] Finding the ordinary methods ineffective, he at length succeeded in separating the bacilli by growing them on a solid medium, a glycerine agar, which was kept at a temperature such that it just remained solid. When grown in this manner the typhoid bacilli form an opalescence spreading through the tube, whereas the B. coli remains confined to the point of inoculation. The centrifuge forms the best means of obtaining the microbes from infected water, but special treatment is required for sewage.

Though he was able to separate the typhoid bacilli by his method more effectively than by Parietti's, Elsner's, or other plans, he found that the difficulty of identifying the true colon bacillus is much greater than is usually supposed. Indeed, he shows that more than a dozen varieties of the latter which fulfil the ordinary tests are really distinct species. Some react with typhoid serum like the typhoid bacillus itself, and there are important differences between the varieties which probably imply different life histories and functions. It has been supposed that their presence indicates fæcal contamination, but if Stoddart's view is correct, this theory is quite untenable. Even the true bacillus coli is not often to be found in the intestines, and the records of its supposed occurrence in many pathological conditions are no longer to be relied on.

He carried on cultivations of many of these species through dozens of generations and found that their characters were maintained unchanged.

Van der Velde and Masius[2] state that some varieties of the typhoid bacillus react much more strongly to typhoid serum than others, while some do not react at all. Similar facts have been observed with regard to the cholera vibrio, the B. coli, and, indeed, in the streptococci absence of agglomeration is generally the case with their proper serum. Van der Velde also confirms Wright's view that dead cultures of typhoid bacilli are agglomerated just as well as living ones, and may be therefore used in Widal's test. G. B. Hunt[3] narrates a case showing the value of the test. The patient had normal temperature, no distension of the abdomen, or diarrhœa, but had been ailing for a month. His blood gave a marked reaction, and after ten days diarrhœa and melœna occurred, followed by a typical relapse of typhoid. A sister was also found to be actually suffering from typhoid at the time.

Delépine,[4] besides detailing his own method of using Widal's reaction, discusses the difficulty of distinguishing by other tests the typhoid bacillus from the various bacilli of the colon type, and finds that at least seven signs must be shown by it, such as the absence of indol and of a rennet ferment. To observe the manner of growth, he relies entirely on the dilution of media, so that he has only a few colonies on a plate at once. He has discarded the use of acids for this purpose, as in Elsner's test. Nevertheless, he mentions that the B. typhosus ceases to grow when there is ·23 per cent. of carbolic in the medium, while the B. coli flourishes till the amount reaches ·27 per cent., which may possibly be used as a means of diagnosis.

The oyster companies are waking up to the injury done to the trade by the infection of a few oyster beds with typhoid excreta, and they are petitioning the Government for the closure of dangerous beds and the inspection of all oysters put on sale so as to restore public confidence in their wares.[5]

**Treatment.**—Elsner Lee[6] instead of the bath allows a spray of cold water to play upon the patient, who is then covered up by a blanket, and the small quantity of water used is afterwards allowed to evaporate. This is repeated every two hours during two days and afterwards at longer intervals. Water is frequently given by the mouth, cold compresses are placed on the abdomen, and rectal injections of warm water are also employed to the amount of two or three litres. No food is given until the patient is convalescent, and drugs or stimulants with the exception of a little digitalis are also withheld. He claims that ulceration and perforation of the bowels are unknown under this treatment, and that the disease can be aborted altogether if taken in the first five days. F. E. Hare[7] gives the results of ten years' experience of the cold bath system in the Brisbane Hospital. The previous mortality had been 14·8 per cent. in 1,800 cases, while with cold baths this fell to 7·5 per cent. in some 1,900 cases. The reduction has not been in preventing hæmorrhage and perforations, but rather in obviating other complications which account for two-thirds of the deaths in typhoid, more particularly, as statistics show, in female patients. J. L. C. Whitcomb[8] instead of the bath tubs uses sponging first with warm and later on with ice-cold water every two or four hours. The sponging occupies thirty to forty-five minutes, the body being gone over several times with brisk rubbing, and when the temperature reaches 105 deg., bags of crushed ice are placed on the abdomen, the thighs, and head. One or two quarts of cold water are drunk in the twenty-four hours. Brandy is given when there is much weakness before each bath, and a hot-water bottle may be placed at the feet. Calomel is employed with a saline at the beginning of the illness, and in smaller doses later on if there is any constipation, as well as salol and strychnia. The diet consists of peptonised milk, beef-broth, and butter milk. No death occurred in ninety-seven cases treated in this way. T. P. Thornley[9] records a mortality of 8 per cent. in 250 patients treated by some form of bathing at the New York Presbyterian Hospital; Thompson, at the Roosevelt Hospital, had a similar mortality in sixty-five cases, with an absence of many of the common complications. From Victoria H. Rabl[10] claims a mortality of only 4·5 per cent. in private practice on 178 patients, and notices that typhoid is there confined to the dry months of the year. He gives a cold bath of fifteen minutes' duration every time the temperature reaches 103 deg. in the rectum. An attendant rubs the body thoroughly all the time the patient is in the water, and a mouthful of some hot drink is given when the patient goes back into his blankets. If he has only one attendant he orders a single dose of an antipyretic at night instead of the baths, and if the baths are commenced early enough he finds the patients nearly always keep up strength enough to step in and out of the bath themselves. Often as many as 100 baths in all are given, and the rapidity of convalescence is remarkable. His system is certainly a remarkable instance of carrying out successfully an elaborate treatment under the greatest difficulties among poor patients. The so-called "abortive treatment" shows a mortality of only 1·4 per cent. in over 4,000 cases collected by the inventor, J. E. Woodbridge,[11] who insists that the system must be carried out in its minutest details, as given in his book with above title.

**Complications.**—F. Plicque[12] regards heart lesions as only the chief causes of death in this disease. Endocarditis, and more often myocarditis, are the commonest forms. Weakening of the first sound, and reduplication of the second at the apex with soft, systolic murmurs, and disturbances of the rhythm or acceleration of the pulse, with pallor, slight œdema, and syncope are among the symptoms. Brandt's baths are the best means of prevention, but in weak patients the cold pack may be substituted, and care must be taken to keep the patient in the horizontal position, and to give hypodermically cardiac stimulants when symptoms of failure appear. Two remarkable cases of death from cardiac failure are reported by Wallace Beatty,[13] though in one only was a bacteriological examination made. In the latter death occurred on the sixth day after jaundice and hæmaturia. The most remarkable fact, however, was that the intestines were normal, and the Peyer's patches and the solitary glands were unaffected. All doubt about this being typhoid without the specific lesions was set at rest by the discovery of typhoid bacilli in the spleen. Similar instances were noted by J. W. Moore in his work on fevers. In the discussion on this case N. Falkiner gave the history of a patient who suffered from a branny desquamation in typhoid. On the feet the skin came off in flakes of the size of a segment of a tapeworm. Any suspicion as to the co-existence of scarlatina was set at rest by the patient accidentally taking that disease shortly afterwards.

**The Plague.**—Yersin[14] states that, after observing the plague at Hong Kong, he injected the bacilli into the veins of horses in repeated doses, and was able to cure mice by the horse serum after they had been infected. When he had obtained a quantity of this anti-toxin, he treated twenty-three patients at Canton and Amoy with it. All of them except two recovered, though the serum had been carried all the way from Paris to China. The quickness of the cure depended on the earliness with which the serum was injected. Yersin[15] also found the bacillus in the soil in a non-virulent form, though when the infection was passed from one guinea-pig to another the virulence was very rapidly augmented. Protection after an attack of plague is of short duration, and this makes it probable that an attenuated virus would have little value as a vaccine. A third method, that of injecting sterilised cultures, might be protective, but the toxins would be fatal where the disease is already present. As to other treatment, T. Cantlie[16] lays stress on a primary calomel purge, followed by a saline. Stimulants, easily-digested and frequent meals during an attack, are important. Cold sponging for the pyrexia, morphia, ammonia, cinchona, and strychnia give good results, but the symptoms must be treated as they arise. All articles used by the patients must be disinfected. The infection is carried very little by the air, except in the dust from the floor and by flies, as Yersin showed. The fæces, urine, and other discharges contain the bacilli, which may also be conveyed by inoculation through wounds, as in the case of Professor Aoyama.[17]

Childe[18] believes that 10,000 persons died from plague in Bombay from August to February, and are actual case of pestis major appeared in Calcutta. The Shropshire Regiment, which was employed two years earlier during the plague in Hong Kong, has ever since been affected with non-syphilitic buboes, and in the blood of some of the men a diplo-bacterium was found.

Haffkine[19] inoculated a number of horses to obtain an anti-toxin, and Yersin took a supply of the serum above described, but the latest accounts, if, indeed, these are reliable, do not show that it was very successful, for the mortality was not appreciably lessened,[20] being about 50 per cent. Haffkine reported some success after preventative inoculations of sterilised cultures of the bacillus, only two attacks occurring in 150 vaccinated persons against eighteen in 170 non-vaccinated, and later on, out of 2,790 vaccinated, no deaths and only three attacks were noticed.[21]

The Government at last insisted on severe measures of segregation and cleansing. Plague Committees were formed in various towns, and active disinfection was carried out with care to avoid irritating native feeling more than necessary.

In Bombay and Karachi the disease is dying out, but some recrudescence had appeared in Bombay in May. On the whole the plague exists in an area which is less than a tenth of India,[22] and shows no sign of spreading into the heart of the country. The cause of the outbreak is still unknown, but the able paper of Dr. A. G. Viegas[23] shows that the insanitary condition of the Port Trust Estate, Bombay, afforded an opening of the most favourable kind. The final report of the results of Yersin and Haffkine's work will be awaited with much nterest.

**Cholera.**—An interesting discovery has been made with regard to the cholera vibrio. Chrysoidin, a compound akin to vesuvin, possesses the power of "clumping" cholera vibrios, just the same as the serum of immune persons does, though no other body, not even those chemically allied to it, has similar properties. In fact, it is an artificial cholera "agglutin" and disinfectant. It may thus act as a prophylactic, though it does not reach the intestines when swallowed, and cannot therefore be used as a remedy. It may be swallowed, however, in teaspoonful doses without harm when in a strength of one in a thousand. Blachstein[24] finds that animals inoculated with cholera cultures to which chrysoidin has been added live, while the control animals die. The question arises whether it might not be a useful remedy if given in keratin capsules, so as to be carried through the stomach unabsorbed.

[1] Analyst, May, 1897. [2] Med. Week, April 2. [3] Clinic Jour., March 24. [4] Med. Chron., March. [5] Brit. Med. Jour., March 6. [6] Epit. Brit. Med. Jour., March 6. [7] Australas. Med. Gazette, March 20. [8] Med. Record, Jan. 30. [9] Med. Record, Jan. 9. [10] Australas. Med. Gazette, Dec. 16. [11] Med. Record, Jan. 16. [12] Indian Lancet, April 1. [13] Dublin Jour. Med. Sc., Feb. 1. [14] Annales de l'Inst. Pasteur, Jan. [15] Practitioner, Jan. [16] Brit. Med. Jour., Jan. 30. [17] Lancet, Feb. 13. [18] B. Med. Jour., Feb. 20. [19] B. Med. Jour., Feb. 23. [20] Lancet, May 15. [21] B. Med. Jour., March 20. [22] Lancet, Ap. 24. [23] Lancet, Ap. 3. [24] B. Med. Jour., Feb. 13.

### DISEASES OF THE LUNGS.—TUBERCULOSIS.

**Aerial Therapy.**—The question whether sanatoriums for consumptives are a danger to the neighbourhood has been discussed in these pages some time back. It is a question to which S. A. Knopf,[1] of New York, has devoted considerable attention, and his recent contribution on the subject is of practical moment, tending to prove that the vicinity of a properly conducted sanatorium for consumptives is absolutely harmless. The author refers to the fact that the most important factors in imparting the disease are the expectoration, the saliva, and other secretions, and points out that in a properly conducted sanatorium patients never expectorate except in a receptacle provided for the purpose—a spittoon or a pocket flask. Those in bed and too weak to make use of the spittoon are provided with moist rags, which are burned immediately after use. The expectoration and other secretions are destroyed before they have a chance to dry and do harm. Napkins and table utensils are boiled or disinfected after each meal. Besides this, a scrupulous cleanliness is observed in all rooms, and the furniture is so arranged that a thorough disinfection may be easily carried out. Knopf further states that repeated microscopical and bacteriological examinations of the dust taken from the rooms of such sanatoriums have proved it to be practically free from bacilli. At Saranac Lake, the great American sanatorium, none of the 20 to 25 attendants have ever developed tuberculosis. The contraction of the disease by physicians, nurses, or employees is almost unknown in these institutions.

But the effect of such sanatoriums upon their surroundings is not a matter of conjecture or of opinion. Knopf brings experience and statistics to our help. In Goerbersdorf, the largest and oldest sanatorium for consumptives in the world, through which some 2,000 patients pass every year, the mortality from tuberculosis among the people of the neighbouring village has decreased in a wonderful degree since the establishment of the institution. Not only has the sanatorium done no harm to the surrounding population, but it has done good, through the example set before the village people by the patients and the sanitary regulations, which direct all attention to the destruction of the bacillus. To uphold these statements he quotes from his thesis the official statistics of the village of Goerbersdorf for 100 years :—

Deaths from Phthisis Pulmonalis.

| | | | |
|---|---|---|---|
| 1790-1799 ... ... ... | 14 | 1840-1849 ... ... ... | 6 |
| 1800-1809 ... ... ... | 5 | 1850-1859 ... ... ... | 7 |
| 1810-1819 ... ... ... | 9 | 1860-1869 ... ... ... | 4 |
| 1820-1829 ... ... ... | 9 | 1870-1879 ... ... ... | 5 |
| 1830-1839 ... ... ... | 8 | 1880-1889 ... ... ... | 5 |

These statistics become still more valuable when one considers that the population of Goerbersdorf has doubled in the last 25 years. Again, the author informs us that recently Dr. Nahm has compiled the statistics of the village of Falkenstein. Here, also, the mortality from pulmonary tuberculosis has been reduced from 18·9 per cent. before the establishment of the sanatorium, to 11·9 per cent. after it was opened. He gives the statistics of Falkenstein in full, as they were published by Dr. Nahm:—

Deaths from Phthisis Pulmonalis.

| Before the Establishment of the Sanatorium. | | After the Establishment of the Sanatorium. | |
|---|---|---|---|
| 1856-1858 ... | 17·2 per 100. | 1877-1879 ... | 17·0 per 100. |
| 1859-1861 ... | 7·7 „ | 1880-1882 ... | 14·6 „ |
| 1862-1864 ... | 22 6 „ | 1883-1885 ... | 6·0 „ |
| 1865-1867 ... | 14·0 „ | 1886-1888 ... | 5·0 „ |
| 1868-1870 ... | 16·7 „ | 1889-1891 ... | 13·9 „ |
| 1871-1873 ... | 21·0 „ | 1892-1894 ... | 15 1 „ |
| 1874-1876 ... | 33·3 „ | | |

Thus, in these sanatoria the patients and their attendants are safer than under the common conditions of home life. Irwin Hance,[2] moreover, has instituted a further study of tuberculous infection of dust, in various localities in New York—tenements, hospitals, out-patient rooms, and public conveyances. As regards the tenements, of 16 pigs inoculated with dust taken from four tenements, where the family had paid attention to the instructions and regulations given them by inspectors, five died of acute infection, and none were infected with tuberculosis. Of 12 pigs inoculated with dust from suspected rooms, the people being careless and dirty, two died of acute infection, one was indicative of tuberculosis, and five were tuberculous; and two tenements out of three were found infected. In Bellevue and Charity hospitals 11 wards were examined, with negative results.

**Guaiacolate of Piperidine.**—This synthetic drug has been employed by Chaplin and Tunnicliffe.[3] In order to put their results with this substance as succinctly as possible, they divide their paper into three parts—(I.) Chemistry, (II.) Pharmacology, (III.) Therapeutics.

I. *Chemistry.*—Piperidine guaiacolate (Schidrowitz*) is a compound formed by the action of piperidine on guaiacol in a suitable solvent, such as benzol or petrolic ether, and it must be regarded as a guaiacolate of piperidine, having the formula $C_5H_{11}NC_7H_8O_2$. Its exact chemical composition is still under investigation. It crystallises in prismatic needles or plates. It is soluble to the extent of 3·5 per cent. in water; it is also easily soluble in most organic solvents. It melts at 79·81 deg. C. It is decomposed into its constituents by mineral acids and alkalies. The chemical property which from a pharmaceutical standpoint is most worthy of note is the relative solubility of this salt. When the insolubility of the carbonate of guaiacol is borne in mind, this becomes emphasised. The solubility is such that gr. x. can be administered in an ounce draught of simple water, or if the specific gravity of the medium be raised by the addition of a little glycerine or mucilage, a dose of gr. xx. to xxx. can be given.

II. *Pharmacology.*—The pharmacology of the guaiacolate of piperidine resolves itself into the pharmacology of guaiacol and piperidine, for it is into these substances that the salt is decomposed, probably not in the acid medium of the stomach, but in the alkaline one of the duodenum. The reason for this assumption is that large doses—ʒ j.—can be given without the slightest eructation of guaiacol. The action of guaiacol is too well known to be discussed here; it acts in the intestine as an antiseptic, in the structures through which it is excreted—for example, the respiratory mucous membrane; it acts also as an antiseptic. They only give now a brief summary of the pharmacology of piperidine, as this subject forms part of a research to be published elsewhere by Tunnicliffe in conjunction with Dr. Lauder Brunton. When hydrochlorate of piperidine, suitably diluted, is injected into the circulation in doses of 0·05 g. pro kilo. body weight, the heart is slowed and the vessels contracted, a considerable rise of blood pressure taking place. When injected under the skin in doses of from 1 to 2 cg. pro kilo., an increase in reflex excitability occurs, so that if the drug is pushed convulsions may develop. Thus in suitable doses piperidine must be regarded as a cardio-vascular tonic and spinal stimulant.

III. *Therapeutics.*—During the last three months an inquiry as to the value of piperidine guaiacolate in the treatment of pulmonary tuberculosis has been carried out at the City of London Hospital for Diseases of the Chest. The patients to whom the drug was given were subjected to close observation, and the effects of the medicine were from time to time noted. In all, 14 cases were placed under observation, of which eight were out-patients and six in-patients. The duration of the observations varied, but as a general statement it may be said that six weeks was the average. In order to test efficiently the value of the drug, cases were chosen more or less haphazard, but the results obtained were gratifying. For further details the reader is referred to the interesting original article, but the authors have formed the following general conclusions: premising that in stating the effects of any new drug upon a given disease, the physician must always guard himself against "over enthusiasm," so often it happens that a new medicine has been reputed to be successful in some affection, and upon fuller trial its effects are found to be trifling, or even nil. Of piperidine guaiacolate it may be generally stated: (1) that experience has shown that it is a perfectly safe drug in doses from 5 to 30 gr. three times a day; (2) that it causes no unpleasant effects; (3) that it is exceedingly well borne by the stomach, and in this respect it is equal to any other derivative of creasote; and (4) that patients while under its influence improved in appetite and general strength.

It will be convenient and instructive to group together and contrast the results obtained from tuberculin, antiphthisin, &c., and from the anti-toxic serums resulting from tuberculous inoculation.

**Tuberculin Injections** as a means of diagnosis have been successfully employed in many districts in the case of cattle, but for the early diagnosis of human tuberculosis they are almost entirely neglected. Grasset and Vedel[4] (Montpelier) have reported to the Paris Academy of Medicine the results they have obtained in 14 patients, results which, in their opinion, may encourage other investigators to employ these injections for diagnostic purposes. They include in their report brief

* The manufacturers are Messrs. Joseph Turner and Co., Queensferry, Flintshire.

notes on each of the cases and a copy of the temperature charts showing the results of the injections, but, though these are interesting and instructive, we can only note here the methods employed and the general conclusions of the authors. We would draw attention to the small dose employed, as Grasset and Vedel obtained their results with much smaller doses, and consequently more certainly harmless doses, than that recommended by Strauss (·01 c.c.): "Following the directions of Professor Nocard, we first dissolved 10 per cent. of tuberculin in a 1:200 solution of carbolic acid in water. A gramme of this, on being mixed with half a litre of boiled water, consequently gave us a 1:5000 solution, each cubic centimetre of this liquid containing 1-5th milligramme of pure tuberculin. Later we used a stronger liquid, mixing 1 gramme of the first solution with 200 cubic centimetres of boiled water, resulting in a 1:2000 solution, each cubic centimetre of which contained ½ milligramme of tuberculin. Our first experiments were made with a dose of 1-10th milligramme, but we soon found that this was too small, the effective dose apparently being from 1-5th to 3-10th milligramme for the first injection, and ½ milligramme for the second. The technique is simple and easy to carry out. The patient is kept in bed and his temperature is taken for two or three days, morning and evening. The injection is made with the usual aseptic precautions, under the skin of the thigh, for instance. The temperature is then taken two (or better three) times a day for two or three days. In no case have we met with abscess, local ill-effects, or eruptions."

After detailing their cases, the authors continue: "The principal conclusion which we have drawn from these facts is the complete innocuousness of hypodermic injections of tuberculin, in the doses and under the conditions indicated. We need not insist upon the fact that there is no danger of rendering thereby a person tuberculous who is not so already, inasmuch as it is a bacterial toxin, and not an attenuated virus. The only possible risk is the aggravation of pre-existing symptoms as the result of reaction in confirmed tuberculous subjects. In this respect, however, we think our 13 cases show conclusively that, when doses not exceeding ½ milligramme are employed, there is no ill-effect whatever. This is an important point established. On the other hand, even with these infinitesimal doses a distinct reaction is obtained in some cases, as is shown by our five first observations. These reactions, therefore, permit of recognising tuberculosis in cases in which the diagnosis of this disease would be difficult, if not impossible to arrive at, without this measure. Such are the two cases enumerated above of pulmonary induration without bacilli in the expectoration (Nos. 4 and 5), two cases of meningitis, one spinal (No. 1), the other cerebral (No. 3), and one case of Addison's disease (No. 2). It must be remembered, however, that in some cases these reactions are slow in making their appearance. Apart from those cases in which the reaction is distinct, and the diagnosis of tuberculosis appears to be certain, judging from what we know of tuberculin, there are others in which the reaction is doubtful. Such are the two cases of pleurisy (Nos. 6 and 7), in which there was but slight or no reaction. Lastly, we find cases in which there is no reaction at all, but this is not by any means to be considered as showing that tuberculosis does not exist. The disease may be cured, as in the case of fungous arthritis (No. 8), or on the contrary, it may be well advanced and unmistakable, with breaking-down of the lungs and numerous bacilli in the expectoration, as in three of the above cases (Nos. 9, 10, and 11). Under such circumstances there is natural impregnation, and small doses, therefore, produce no effect; moreover, the diagnosis is clear without this test. For small doses of tuberculin to produce any effect, it appears to be necessary that the lesions be but slightly affected by the microbial toxin. The same fact is mentioned in the instructions for the tuberculin test in cattle. Lastly, it has been pointed out that the reaction may also be obtained in syphilis, leprosy, and actinomycosis. As a matter of fact, there was slight reaction in a non-tuberculous patient who had had syphilis (No. 13). This sign, therefore, is of no value for diagnostic purposes when one has to do with a syphilitic (leprous or actinomycotic) subject. On the whole, the results of our researches are sufficiently encouraging to suggest that the Academy may some time be enabled to issue instructions for the diagnosis of human tuberculosis by tuberculin, as it has been asked to do for the diagnosis of tuberculosis in cattle."[4] Another valuable article by Dr. Sandberg, of Bergen (B.M.J., Oct. 17, 1896), deals with the use of tuberculin for surgical diagnosis.

[1] Med. Rec., Oct. 3, 1896. [2] Ibid., Feb. 14, 1897. [3] Brit. Med. Journ., Jan. 16, 1897. [4] Med. Week, Feb. 28, 1896.

